# Short-Term and Long-Term Outcomes of Robotic Gastrectomy for Gastric Cancer: A Single-Center, Single-Arm Prospective Study

**DOI:** 10.7759/cureus.79063

**Published:** 2025-02-15

**Authors:** Nobuhiko Kanaya, Shinji Kuroda, Yoshihiko Kakiutchi, Hajime Kashima, Satoru Kikuchi, Masahiko Nishizaki, Shunsuke Kagawa, Toshiyoshi Fujiwara

**Affiliations:** 1 Department of Gastroenterological Surgery, Okayama University Graduate School of Medicine, Dentistry, and Pharmaceutical Sciences, Okayama, JPN; 2 Department of Surgery, Tsuyama Chuo Hospital, Tsuyama, JPN

**Keywords:** gastric cancer, long-term outcome, prospective study, robotic gastrectomy, short-term outcome

## Abstract

Background: Robotic gastrectomy (RG) has emerged as a promising approach for gastric cancer (GC) treatment, offering advantages such as enhanced dexterity, improved visualization, and increased precision. However, its widespread adoption remains limited due to technical complexity, high costs, limited applications, and insufficient evidence.

Methods: We conducted a single-center, prospective study to evaluate the safety and feasibility of RG, including robotic total gastrectomy (RTG), robotic proximal gastrectomy (RPG), and robotic distal gastrectomy (RDG) with D1+ or D2 lymphadenectomy, in clinical stage I/II GC. The primary endpoint was the incidence of intraoperative and postoperative complications, while the secondary endpoints included surgical outcomes and long-term prognosis.

Results: Seven patients were enrolled. No intraoperative complications or conversions to open surgery occurred. The primary endpoint was met, with no major postoperative complications. RTG had a longer operative time and more lymph nodes dissected than RDG and RPG. The median postoperative hospital stay was 10 days. Recurrence was observed in two cases, one of which achieved long-term survival without chemotherapy.

Conclusion: Our findings demonstrate the safety and feasibility of RG for early and advanced GC. Further multicenter studies with larger cohorts are needed to establish its oncological benefits and cost-effectiveness, facilitating broader clinical adoption.

## Introduction

Robotic surgery has transformed the field of minimally invasive surgery by offering enhanced dexterity, improved visualization, and exceptional precision [1]. Among these innovations, robotic gastrectomy (RG) has emerged as a promising approach for the treatment of gastric cancer (GC), particularly in East Asia, where the disease burden is among the highest globally [2]. Compared to conventional laparoscopic gastrectomy (LG), RG is expected to provide several potential advantages, including reduced surgeon fatigue, improved ergonomics, and increased surgical precision, particularly in challenging procedures such as lymphadenectomy and reconstruction.

A multi-center, prospective, single-arm phase II study initiated in Japan in 2014 to evaluate the safety and feasibility of RG for clinical stage I/II GC confirmed its potential clinical advantages, including reduced morbidity compared to LG, and RG for GC was approved for health insurance coverage in Japan in 2018 [3,4]. However, despite these advancements, the widespread adoption of RG in Japan has been relatively slow. Several factors have contributed to this trend, including the high initial and maintenance costs of robotic systems, the steep learning curve requiring extensive specialized training, and concerns regarding safety and long-term oncological outcomes during the early phases of implementation [5].

At Okayama University Hospital, the da Vinci Surgical System (Intuitive Surgical, Inc., Sunnyvale, CA, USA) was introduced in 2010, and our first RG procedure was performed in 2011, beginning with robotic distal gastrectomy (RDG) with D1+ lymphadenectomy for early GC patients. In our initial 10 cases, we experienced no intraoperative complications or conversion to open surgery, with one case of postoperative anastomotic bleeding, demonstrating the safety and feasibility of RDG with D1+ lymphadenectomy for early GC. Encouraged by these outcomes, we aimed to expand the indications for RG to more advanced and complex procedures. Specifically, we sought to evaluate the safety and feasibility of robotic total gastrectomy (RTG) and robotic proximal gastrectomy (RPG) for upper GC, as well as RG requiring D2 lymphadenectomy for advanced GC. To achieve this, we conducted a single-center prospective study to assess surgical outcomes and technical feasibility across these more challenging indications.

## Materials and methods

Study design and participants

This study was designed as a single-arm, non-comparative, open-label, single-center, prospective clinical study. This study conformed to the provisions of the Declaration of Helsinki, and the protocol was approved by the Okayama University Hospital Institutional Review Board (Approval no. 1612-010). The University Hospital Medical Information Network (UMIN) clinical trial registration number was 000024297.

The inclusion criteria were as follows: 1) histologically diagnosed with GC, 2) diagnosed with clinical stage I or II according to the Japanese classification of gastric carcinoma (3rd English edition) [6], 3) deemed curatively resectable by RDG, RTG, or RPG with D1+ or D2 lymphadenectomy according to the Japanese gastric cancer treatment guidelines 2014 [7], 4) ineligible for endoscopic treatments such as endoscopic mucosal resection and endoscopic submucosal dissection, and 5) age ≥40 years. The exclusion criteria were as follows: 1) treated with neoadjuvant chemotherapy and 2) ineligible due to mental disorder, pregnancy, or other conditions. These inclusion and exclusion criteria were based on a multi-institutional prospective study that was ongoing at the time [3]. All patients who met the inclusion/exclusion criteria were invited for screening, and written informed consent was obtained from the patient by an investigator before the intervention.

The target sample size was set as seven, which was the number of cases required for Okayama University Hospital to fulfill the facility requirements necessary for participation in a multi-institutional prospective study of robotic gastrectomy [3].

Intervention

RDG, RTG, or RPG with D1+ or D2 lymphadenectomy was performed using the da Vinci S and Xi Surgical System with double bipolar method [8] by a surgeon certified by the Board Certified Surgeon in Gastroenterology, the Endoscopic Surgical Skill Qualification System, and the da Vinci System Training. An assistant used energy devices such as ultrasonic laparoscopic coagulation shears and laparoscopic staplers for the S System, while the operator primarily used robotic devices for these purposes in the Xi System. D2 lymphadenectomy was performed following the same principles as previously reported for LG with D2 lymphadenectomy [9]. Regarding the reconstruction method for each procedure, Billroth-I (B-I) or Roux-en-Y (RY) was selected for RDG, RY was chosen for RTG, and esophagogastrostomy using the double-flap technique (DFT), which has a strong anti-reflux potential [10-12], was employed for RPG. If robotic surgery was deemed difficult due to complications such as massive bleeding, the procedure was converted to open surgery.

Endpoints

The primary endpoint was the incidence of intraoperative and postoperative complications. The secondary endpoints included surgical outcomes, such as total operation time, console time, blood loss, presence or absence of open conversion, and the number of dissected lymph nodes; short-term postoperative outcomes, such as postoperative length of stay; and long-term outcomes, including prognosis.

Information was obtained from the medical record regarding the following factors: patient background including age, sex, body mass index (BMI), American Society of Anesthesiologists physical status (ASA-PS), medical history, tumor location, and clinical stage; short-term surgical outcomes including procedure, extent of lymph node dissection, reconstruction method, operation time, console time, blood loss, number of dissected lymph nodes, postoperative complications, and length of hospital stay; postoperative pathological findings including histology, pathological stage, and residual tumor; presence or absence of adjuvant chemotherapy; and long-term prognosis regarding recurrence and survival. Information regarding tumor location, clinical and pathological classification, histology, and residual tumor was described according to the Japanese classification of gastric carcinoma (3rd English edition) [6]. Intraoperative and postoperative complications were recorded according to the Clavien-Dindo (CD) classification [13]. All patients were followed up regularly at one month, six months, or one year after surgery.

## Results

Seven patients were enrolled in this study based on the inclusion and exclusion criteria as originally planned between May 2017 and June 2018. Patient background is shown in Table 1. The median age was 74 years old (range, 64-77) and the male-to-female ratio was 6:1. The median BMI was 24.2 kg/m2 (range, 23.2-27.5). Based on medical history, four and three patients were classified as ASA-PS 1 and 2, respectively. The tumor was located in or had spread to the upper third of the stomach in five cases. Five and two cases were diagnosed with clinical stage I and II, respectively.

**Table 1:** Patient background BMI, body mass index; ASA-PS, American Society of Anesthesiologists physical status; U, Upper third; M, Middle third; L, Lower third. * described according to the Japanese classification of gastric carcinoma (3rd English edition) [6].

Case	Age	Sex	BMI, kg/m^2^	ASA-PS	Medical history	Tumor location^*^	cTNM^*^	cStage^*^
1	77	Female	23.2	1	Atrial fibrillation	MU	cT1bN0M0	IA
2	75	Male	26.2	1	None	UM	cT3N1M0	IIB
3	69	Male	27.5	1	Dyslipidemia	U	cT3N0M0	IIA
4	76	Male	23.5	2	Cerebral infarction	M	cT1bN0M0	IA
5	66	Male	23.5	1	Hypertension	L	cT2N0M0	IB
6	74	Male	24.2	2	Diabetes, Sick sinus syndrome	U	cT1bN0M0	IA
7	64	Male	26.7	2	Hypertension	MU	cT1bN0M0	IA

Short-term surgical outcomes are shown in Table 2. DG, TG, and PG were performed in two, four, and one cases, respectively, and D1+ and D2 lymphadenectomy were performed in four and three cases, respectively. Regarding reconstruction method, B-I and RY were selected in one case each in RDG, while RY was chosen for all RTG cases, and DFT was employed for one case of RPG. Regarding concomitant procedures, cholecystectomy was performed in Case 1, and splenectomy was not performed in any of the cases. Total operation time tended to be longer in RTG/RPG (mean, 531 min) than RDG (mean, 424 min), reflecting a longer console time in RTG/RPG (mean, 418 min) than RDG (mean, 338 min). The median blood loss was 100 mL (range, 0-650). No conversion to open surgery or intraoperative complications were observed. The number of dissected lymph nodes was higher in RTG (mean, 47) than in RDG (mean, 20) and RPG (mean, 21). The median length of postoperative hospital stay was 10 days (range, 9-11), and the postoperative course was uneventful in all cases, with no postoperative complications or deviations from the clinical pathway.

**Table 2:** Short-term surgical outcomes TG, Total gastrectomy; DG, Distal gastrectomy; PG, Proximal gastrectomy; D, Extent of lymph node dissection; RY, Roux-en-Y; B-I, Billroth I; DFT, Esophagogastrostomy using double-flap technique; LNs, Lymph nodes.

Case	Procedure	D	Reconstruction	Total Operation time (Console time), min	Blood loss, mL	Conversion to open surgery	Intraoperative complications	Number of dissected LNs	Postoperative complications	Length of stay, day
1	TG	1+	RY	495 (371)	50	None	None	54	None	11
2	TG	2	RY	554 (462)	550	None	None	47	None	11
3	TG	2	RY	592 (465)	650	None	None	48	None	11
4	DG	1+	RY	413 (329)	0	None	None	19	None	9
5	DG	2	B-I	436 (347)	100	None	None	21	None	10
6	TG	1+	RY	485 (394)	20	None	None	38	None	9
7	PG	1+	DFT	491 (398)	310	None	None	21	None	9

Pathological findings and long-term outcomes are shown in Table 3. The main histological types were tubular adenocarcinoma (tub) in four cases and poorly differentiated adenocarcinoma (por) in three cases. The pathological stage was higher than the clinical stage in four cases, one of which (Case 3) was diagnosed with pathological stage IV due to positive peritoneal lavage cytology, resulting in R1 resection. In this Case 3, after confirming positive cytology through intraoperative rapid cytology, we performed total gastrectomy with D2 lymphadenectomy as a proposed procedure in accordance with the guidelines 2014 [7]. Adjuvant chemotherapy was administered in four cases, although in two of these cases (cases 1 and 5), adjuvant chemotherapy was not strictly indicated according to the guidelines; however, S-1 adjuvant chemotherapy was administered with the patient’s informed consent.

**Table 3:** Pathological findings and long-term outcomes R, Residual tumor; CY, Peritoneal lavage cytology; tub, tubular adenocarcinoma; por, poorly differentiated adenocarcinoma * described according to the Japanese classification of gastric carcinoma (3rd English edition) [6].

Case	Histology^*^	pTNM^*^	pStage^*^	R^*^	Adjuvant chemotherapy	Recurrence	Recurrence site	Survival	Survival period, month
1	por2	pT1bN2M0	IIA	0	+	-		alive	37
2	por2	pT4aN3aM0	IIIC	0	+	+	gallbladder	dead	32
3	tub2	pT4aN0M1	IV	1 (CY)	+	+	peritoneum	alive	88
4	tub2	pT1bN0M0	IA	0	-	-		alive	60
5	tub2	pT1bN3aM0	IIB	0	+	-		alive	60
6	tub1	pT1aN0M0	IA	0	-	-		dead	6
7	por	pT1bN0M0	IA	0	-	-		alive	60

Recurrence was observed in two cases. One (Case 2) had gallbladder metastases, diagnosed 27 months after surgery following a cholecystectomy originally planned for acute cholecystitis, and this patient died 32 months after the initial surgery. Another (Case 3), who underwent R1 resection due to CY1, was diagnosed with peritoneal recurrence six months after surgery via computed tomography during S-1 adjuvant chemotherapy. This patient switched from adjuvant chemotherapy to systemic chemotherapy for recurrent GC according to the guidelines, and continued chemotherapy for 27 months. This patient is currently alive 88 months after surgery without chemotherapy. The other five patients without recurrence completed regular follow-ups as planned three to five years after surgery, except for one patient (Case 6), who died of an unknown cause six months after surgery.

## Discussion

One of the primary advantages of RG over LG is its superior dexterity and enhanced visualization, which allow for more precise dissection and reconstruction. Our study supports this assertion, as we observed no intraoperative complications or conversions to open surgery, and all procedures were completed successfully. Regarding the short-term outcomes, many studies have reported the clinical benefits of RG compared to LG, such as reduction of postoperative complications including anastomotic leakage and pancreatic fistula [14-16]. To establish more robust evidence, a multi-center prospective study with a large cohort to investigate the superiority of RG over LG in the Japan Clinical Oncology Group (JCOG1907) is currently ongoing [17].

As postoperative recovery was uneventful in all cases, with no complications or deviations from the clinical pathway in our study, improvement of short-term outcomes reflects the postoperative course. The median postoperative hospital stay was 10 days, which is comparable to previous reports on RG and shorter than previous reports on LG [18,19]. On the contrary, the operation time was long and the blood loss was high compared to previous reports [5,14,16,20]. In general, RG tends to have a longer operation time and less blood loss compared to LG. Although the cases in this study are from an early phase of experience, these challenges need to be addressed, as we have overcome them through the standardization of the procedure and the training system in LG [19]. While robotic surgery has been associated with reduced morbidity and faster recovery times, the benefits must be weighed against the economic burden and resource allocation. The high initial and maintenance costs of robotic systems remain a significant barrier to widespread adoption, and further cost-effectiveness analyses are needed to justify broader implementation. Robotic surgery may also have the advantage of reducing labor cost, as it can be performed with only one assistant.

Another key finding of our study was the number of dissected lymph nodes, which was higher in RTG than in RDG and RPG. This difference may be attributed to the range and complexity of lymphadenectomy in RTG, particularly in cases requiring D2 dissection, although this also applies to open and laparoscopic surgery and is not limited to RG. The ability of the robotic system to provide stable and magnified three-dimensional visualization likely contributes to the precise nodal dissection. In comparison between RG and LG regarding the number of dissected lymph nodes, a previous study reported that the number of dissected lymph nodes was significantly higher in RG than LG, especially in TG and PG [16], while another study reported no significant difference between RG and LG [14]. However, despite these technical advantages, the long-term oncological benefits of RG compared to LG remain to be fully established, although several retrospective studies reported no significant oncological benefits of RG compared to LG [15,20,21]. In our study, recurrence was observed in two cases. One patient (Case 2) had gallbladder metastases 27 months postoperatively and succumbed to the disease 32 months after initial surgery. Another patient (Case 3) experienced peritoneal recurrence at six months postoperatively but responded well to systemic chemotherapy, achieving long-term survival of 88 months without further treatment. These findings highlight the need for vigilant postoperative monitoring, particularly in patients at high risk for recurrence, and underscore the importance of individualized adjuvant therapy strategies.

Despite the acceptable results, our study has several limitations. First, the sample size was very small, limiting the generalizability of our findings. While our study met the required number of cases for Okayama University Hospital’s participation in a multi-institutional study, larger-scale research is necessary to validate our results. Second, this study was conducted by only one experienced surgeon trained in robotic surgery. The outcomes may not be directly applicable to less experienced surgeons, emphasizing the need for structured training programs and standardized protocols to ensure the safe dissemination of RG techniques.

## Conclusions

Our study supports the safety and feasibility of RG for gastric cancer, including RTG, RPG, and RDG with D1+ or D2 lymphadenectomy. The absence of intraoperative complications, acceptable operation times, and favorable postoperative courses suggest that RG is a viable option for both early and advanced GC. However, further multicenter studies with larger sample sizes and longer follow-up durations are required to establish the definitive oncological benefits and cost-effectiveness of RG. Continued advancements in robotic technology, coupled with refined surgical techniques and training programs, will likely contribute to the broader adoption of RG in the future.
